# Simple Color Calibration Method by Projection onto Retroreflective Materials

**DOI:** 10.3390/jimaging8090239

**Published:** 2022-09-03

**Authors:** Yusuke Nakamura, Takahiko Horiuchi

**Affiliations:** Graduate School of Science and Engineering, Chiba University, Yayoi-cho 1-33, Inage-ku, Chiba 263-8522, Japan

**Keywords:** retroreflective materials, color calibration, projector, AIC

## Abstract

Retroreflective materials have the property of directional reflection, reflecting light strongly in the direction of the light source, and have been used for road traffic signs. In recent years, retroreflective materials have been used in entertainment and industrial technologies, in combination with projection mapping technology. In general, color calibration is important when projectors are used to control reflected colors. In this study, we investigated a simple color calibration method for retroreflective materials with a 3D shape under the condition that they are observed in the same direction as the light source. Three types of retroreflective materials with different reflective properties were used. First, to measure the reflective properties of each reflective material, the reflective material was fixed to a flat plate and rotated, while the reflected light was measured in the same direction as the light source. It was then confirmed that the reflected light intensity varied smoothly with angular change, and appropriate measurement angles were investigated based on the AIC criterion, aiming to interpolate the reflectance characteristics from a small number of measurement angles. Color calibration was performed based on the reflectance characteristics obtained from the derived measurement angles, and the experiments showed that good color calibration was possible with fewer measurements.

## 1. Introduction

Retroreflective materials can reflect incident energy back towards its source, regard-less of the direction of incidence. Many retroreflective films are available, aimed primarily at the road, rail, and air transport industries, for signs and route markers that are highly visible in all weather conditions. Recently, retroreflective materials have been applied in entertainment and industrial technology by combining retroreflective materials and projection mapping technology. An example is retroreflective projection technology (RPT), which was studied by Tachi et al. [1,2,3,4,5]. RPT is realized by using retroreflective materials as the projection target and setting the light source and observer at conjugate positions using half mirrors. Transparent cockpits [6], which make the passenger seat and door areas transparent, and transparent Prius [7], which makes the back seat of a car transparent, have been proposed. Bhardawaj et al. [8] briefly discussed optical camouflage using RPT and its potential for entertainment and industrial applications. However, brightness and color reproduction in these image displays are not sufficient. This may be attributed to the fact that the retroreflective performance by the retroreflective material varies with the angle of incidence, and the intensity of the reflected light varies slightly because the screen in the projection area is not flat. Therefore, color calibration with a suitable projection angle is important when controlling reflective color using RPT, but few color calibration methods have been studied for retroreflective materials.

In general, a calibration process is required for each projection target and projection device, and it is desirable that the process be as easy as possible to implement. In addition, it is necessary to determine the reflective characteristics of the projection target to perform color calibration. If the incidence and observation angles are fixed, color calibration can be completed under these conditions by deriving a look-up table (LUT) in the same way as for ordinary reflective objects. However, since the reflection intensity of retroreflective materials varies irregularly with angle, the LUT must be derived for all angular conditions. To obtain the reflective properties of directional reflective objects, such as retroreflective materials, goniometric measurements must be performed at different angles of incidence and observation [9,10]. Therefore, such measurements often require an enormous amount of time. The authors focused on the fact that retroreflective materials are often used when the light source and observer are conjugate and the optical axes are coincident or in the same direction, as in RPT, and thought that the measurement of reflectance characteristics and color calibration based on them can be simplified by limiting the conditions to the same direction as the angle of incidence and angle of observation. By limiting the conditions to the same direction of the incidence angle and observation angle, the measurement of reflection characteristics and color calibration based on them could be simplified.

Based on the above background, the purpose of this study is to propose a simple color calibration method for projectors in RPT for retroreflective materials with a known 3D shape, under the condition that they are observed in the same direction as the light source. As mentioned above, a color calibration process is required for each projection target and device. In other words, the reflectance characteristics must be measured for color calibration when the projection target or device is changed. In this study, we propose a color calibration method for the latter case. Specifically, when a new projection device is used, we aim to obtain the reflection characteristics of a projection from a projector onto a retroreflective material of known structure with a small number of measurements and to perform overall color calibration using these data. The effectiveness of the proposed method is verified through actual projection experiments.

## 2. Measurement of Reflectance Characteristics

To confirm whether the reflective characteristics differ depending on the difference in the surface structure of the retroreflective materials, measurements were performed on three types of retroreflective materials in this study. The characteristics of the retroreflective materials used, measurement environment, and measured reflective properties are described in this section.

### 2.1. Measured Objects

**Figure 1:**
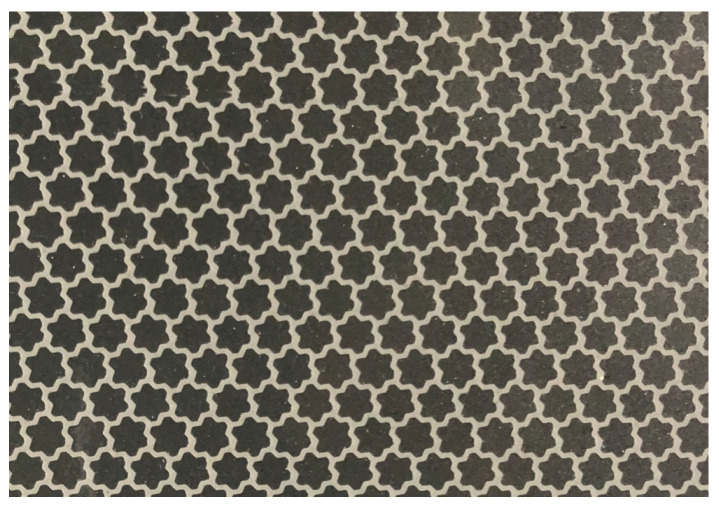
Close-up photograph of a glass bead type material.

Retroreflective materials can be classified into prism and microscopic glass bead types, depending on the structure used for retroreflective reflection. The prism type has a structure in which a prism shaped like a right-angled triangular pyramid is bonded to the surface of the sheet, and the light that enters the sheet is repeatedly reflected by the prism surface, causing retroreflective reflection. The glass bead type has a structure in which minute glass beads with a high refractive index are laid on the sheet surface, and the incident light is refracted by the glass beads, reflected by the reflective layer, and refracted again by the glass beads to cause retroreflective reflection. Owing to this structure, the glass bead type has fewer restrictions on the direction of light incidence than the prism type. Therefore, in this study, we limited the measurement of reflectance characteristics to the glass bead type. Figure 1 shows a close-up image of the glass bead type.

Glass bead retroreflective materials can be further classified into three types according to their surface structure. The three types are the “open lens type”, in which the glass beads are exposed; the “enclosed lens type”, in which the surface of the open lens type is covered with a transparent, smooth resin layer; and the “encapsulated lens type”, in which the surface film is floated by cell-like bridges to suppress the degradation of performance due to light refraction. The open lens type is mainly used for clothing and shoes, whereas the enclosed lens type is used for license plates, traffic cones, and other applications where durability is required. The encapsulated lens type is mainly used in situations where high-intensity reflection is required, such as signage and life vests. To investigate the differences in reflective properties due to differences in surface structure, this study measured the reflective properties of three types of Unitika Sparklite Corporation’s “open lens type: MT-801 White”, “enclosed lens type: 701 White”, and “encapsulated lens type: Sun Life 2C”.

### 2.2. Measurement Environment

**Figure 2:**
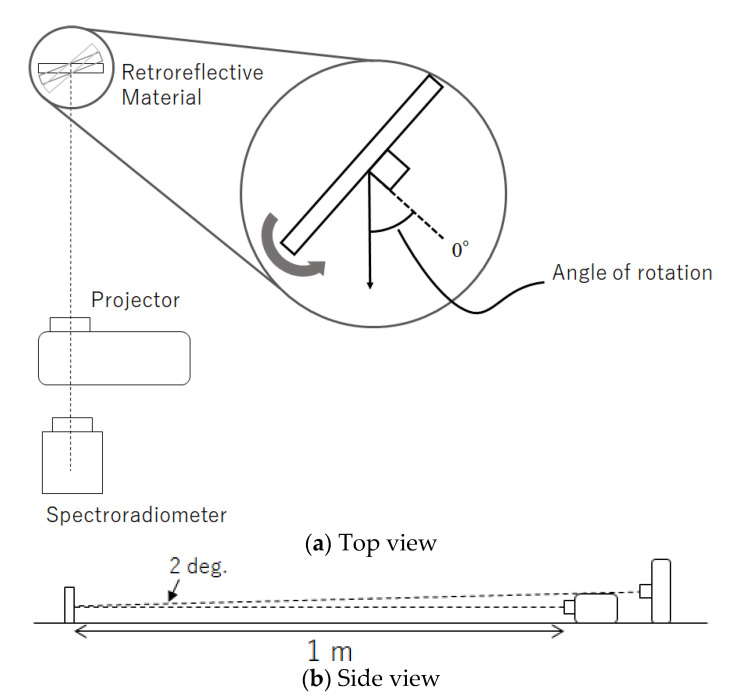
Measurement environment.

Three types of retroreflective materials were fixed to a 20 cm square flat plate, and the plate was rotated by controlling its rotation with a rotating stand to measure different angles. We used a rotation motorized stage OSMS-60YAW (Sigma Koki Co., Tokyo, Japan) for control. The projection distance from the light source to the retroreflective material was 1 m. In this study, the light source and observer were in the same direction, so the measurement device was placed behind the projector. The optical axes of the projector and the measurement device were aligned horizontally and vertically angled at 2°. Figure 2 illustrates the measurement environment. Because the object was rotated for measurement, the distance from the background was set to 20 cm to avoid contact with the object. Measurements were taken in a dark room, and the background was black wallpaper in the dark room to avoid the influence of ambient reflected light. The angle of rotation was defined as the angle between the measurement device and the line perpendicular to the flat plate, to which the retroreflective material was fixed, with the perpendicular line at 0° in front of the plate. There were 12 rotation angles: 0°, 15°–60° in 5° increments, and 75°. A total of 256 colors (64 RGBW colors (each pixel value from 1 to 15 in increments of 1 and from 1 to 255 in increments of 5) were used for projection using a projector EMP-TW1000 (Seiko Epson Corporation: Nagano, Japan). A spectroradiometer CS2000 (Konica Minolta, Inc.: Tokyo, Japan) was used to obtain the CIE XYZ values of reflected light.

### 2.3. Reflection Characteristics

**Figure 3:**
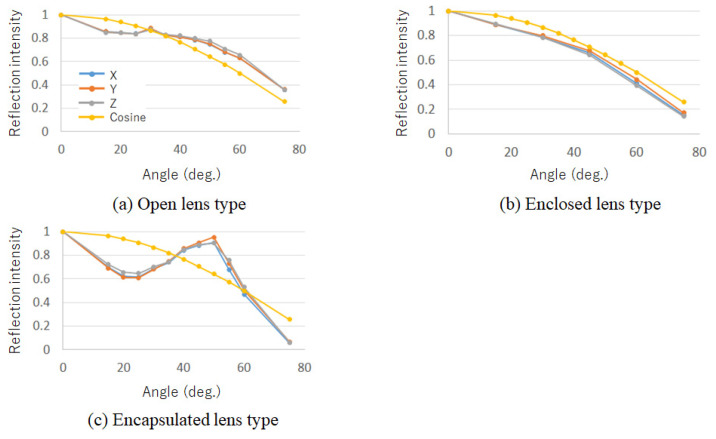
Reflection intensity per angle of rotation. For comparison, the intensity change according to cosine is superimposed.

Figure 3 shows the reflection intensity at each angle, normalized by the frontal (0°) reflection intensity. For comparison, a cosine law graph assuming Lambert reflection in general diffuse reflection is also shown. As a result of the measurement in Figure 3b, the encapsulated lens type has characteristics similar to Lambert reflection, and that color calibration can be performed based on the cosine law. However, as shown in Figure 3a,c, the open and encapsulated lens types have characteristics different from Lambert reflection.

## 3. Color Calibration Method

In this section, we discuss color calibration based on the reflection characteristics obtained in Section 2. Here, because the results of Section 2 confirm that color calibration of the enclosed lens type is possible based on the cosine law, the color calibration of open and encapsulation lens types are discussed. In general, color calibration can be achieved by multiplying the output signal with the inverse function of the reflection intensity characteristic with respect to the angle of projection onto the projected object. However, it is not realistic to measure reflection characteristics that change in a complex manner for all angles, and to have the data as a look-up table (LUT). Therefore, we focused on the fact that the reflection intensity of the open and encapsulated lens types varies smoothly with angular variation based on the results of Section 2 and attempted to generate a color calibration LUT by expressing the reflection characteristics using a small number of measurement angles (hereinafter referred to as “reference points”). Specifically, we seek measurement points that can represent reflection characteristics with high accuracy using quadratic B-spline interpolation of a small number of reference points. The following describes the method for determining the number of reference points and generating a color-calibrated projection light.

### 3.1. Method of Determining the Number of Reference Points

In general, there is a tradeoff between the number of reference points and the accuracy of the representation. In other words, measurements with fewer reference points are desirable, but a decrease in the number of reference points leads to a decrease in accuracy. Therefore, the Akaike information criterion (AIC) [11] was used to determine the measurement angle as the norm for model evaluation to minimize the number of reference points and interpolation accuracy. The AIC is generally given by the following equation:

(1) AIC=−2logL+2k

where *L* is the maximum likelihood of the model being evaluated and *k* is the number of estimated parameters in the model. When comparing AICs among models, it was shown that selecting the smallest model will select a good model in many cases. Here, models with different numbers of reference points expressed by interpolation were compared using the AIC to determine the optimal number of reference points. As interpolation methods, we applied linear interpolation, Lagrangian interpolation, and nth-order B-spline interpolation based on the results of the reflection characteristics obtained in Section 2, particularly for the encapsulated lens type, which has complex characteristics such as repeated increases and decreases, and confirmed that good results were obtained with quadratic B-spline interpolation. Therefore, in this study, quadratic B-spline interpolation was used for color calibration as an interpolation method for reflection characteristics.

**Figure 4:**
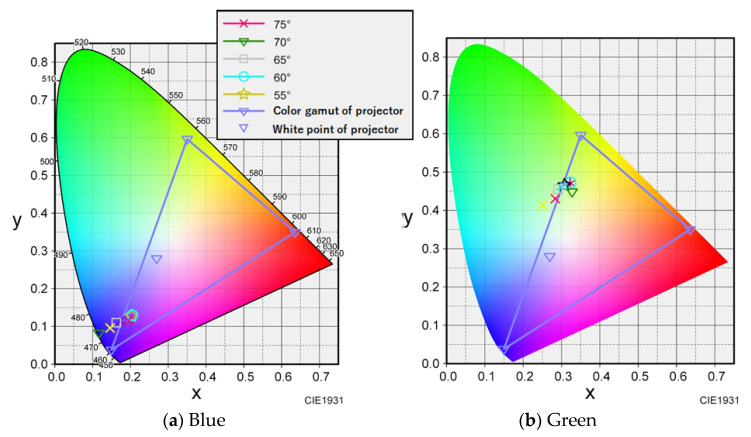
Color gamut of the projector and color-calibrated value for each angle.

To determine the number of reference points, 12 angles were measured from 0° to 75°. Color calibration was performed on the measured data from each angle, and it was confirmed that the larger the measurement angle of the encapsulated lens type, the more the output data tended to be outside the gamut of the projector. Specifically, it was found that some colors could not be reproduced when the measurement angle was greater than 60°. Figure 4 shows the color calibration data for each angle for blue and green, including the projector gamut and colors that could not be reproduced. For the open lens type, the projector was found to fall within the gamut of the projectors in the range of 0°–75°. Therefore, we changed the range of the data used for the open and encapsulated lens types. The open lens type used data in the range of 0° to 75°, whereas the encapsulated lens type used data in the range of 0° to 55°, considering that the larger the angle, the more likely the data would be outside the color gamut.

To select an appropriate number of reference points for each AIC, we created models with different numbers of reference points by selecting a few points from each measurement angle for comparison. The procedure for applying the AIC is as follows:

1. The number of reference points compared ranged from 3 to 6. For the open model, the reference points were selected from the range of 0°–75°, and for the capsule model, from 0°–55°, with both ends (0° and 75° or 55°) fixed and equally spaced between the two angles.
2. Using the data for each reference point, a quadratic B-spline function was used to interpolate the reflection intensity data (CIE XYZ) in 1° increments for open and encapsulated ranges.
3. The difference between the interpolated data in each range obtained in this study and the data obtained in the measurement was calculated for the reflection intensity data, and the maximum likelihood *L* in Equation (1) was used to calculate the AIC. Specifically, assuming that the approximation errors at each angle were independent of each other and normally distributed with mean zero and variance $\sigma^{2}$, the maximum likelihood *L* was calculated as follows:
  (2) $$L={\left(\frac{1}{\sqrt{2\pi }\hat{\sigma }}\right)}^{N}e^{-Q/\left(2{\hat{\sigma }}^{2}\right)},$$
  where *Q* means the sum of squares of the approximation error, *N* represents the number of reference data used to compute the approximation error (*N* = 76 for the open lens type and *N* = 56 for the encapsulated lens type), and ${\hat{\sigma }}^{2}$ is the maximum likelihood estimator of $\sigma^{2}$, using the relationship ${\hat{\sigma }}^{2}=Q/N$.

As explained above, what we are evaluating with the AIC by *k* measured angles is whether the model is able to follow the changes in CIEXYZ reflection intensity in Figure 3 for each incidence angle. Table 1 presents the AIC for each number of reference angles *k* for the open and encapsulated lens types. Table 1 shows that the AIC is smallest when the number of reference points is four for both the open and encapsulated lens types, indicating that the reflection characteristics can be represented most efficiently.

### 3.2. Generation of Color Calibration Projection Light

Table 1 confirms that the most suitable number of reference points for both the open and encapsulated lens types is four. Therefore, the AIC was derived from the four reference points to examine the relationship between the optimal combination of reference points and AIC among the measured angles. Because both ends are fixed, it is necessary to select two points between them. For the open lens type, 2 points were selected from 10 points between 15° and 60° out of 12 points between 0°, 15°, 60° (in increments of 5°), and 75° because 0° and 75° were used at both ends. For the encapsulated lens type, two points were selected from eight points between 15° and 50° out of nine points between 0°, 15°, and 55° (in increments of 5°) because 0° and 55° are used at both ends. The AIC was obtained for each candidate combination of the reference points selected in this manner.

As a result, it was found that the AIC differed greatly depending on the reference points selected. To confirm that the reference point combinations selected by the CIC rep-resent the reflection characteristics with high accuracy and allow for correct color calibration, verification experiments were conducted using a few reference points for each open and encapsulated lens type. Several combinations of reference points were selected from those with small to large AIC values to compare the results of the verification experiment with those of the AIC. Specifically, six open lens types were selected: (0, 20, 25, 75), (0, 25, 30, 75), (0, 25, 40, 75), (0, 25, 45, 75), (0, 25, 50, 75), and (0, 40, 50, 75); and six encapsulated lens types were selected: (0, 20, 40, 55), (0, 20, 45, 55), (0, 20, 50, 55), (0, 25, 40, 55), (0, 25, 45, 55), and (0, 25, 50, 55). Color calibration was performed by multiplying the reflection characteristics (ratio of the reflection intensity at each angle to 0°) obtained by quadratic B-spline interpolation of the reference points for the above combinations by the LUT for color calibration at 0°.

## 4. Verification Experiment

In the previous section, we performed simple color calibration for the open and encapsulated lens types by expressing the reflection characteristics of the four reference points using quadratic B-spline interpolation. This section describes the experimental environment, results, and discussion of the verification experiment conducted to confirm the effectiveness of the color calibration projection light created in Section 3 and to compare it with the AIC results.

### 4.1. Experimental Environment

Experiments were conducted in a dark room. The arrangement of the equipment was the same as that in Figure 2, and a digital camera (Canon EOS-1Ds Mark III) was installed at the position of the measurement device. In the camera calibration, at first, we measured the spectral responses of the digital camera using a monochromator SPG-100 (Shimadzu Corporation: Kyoto, Japan) and a spectroradiometer CS2000 (Konica Minolta, Inc.: Tokyo, Japan). Then, by least-squares approximation of the response function to the CIE 1931 2-degree XYZ color matching functions, we derived a 3 × 3 matrix that converts 14-bit RGB raw camera data to CIE XYZ tristimulus values.

**Figure 5:**
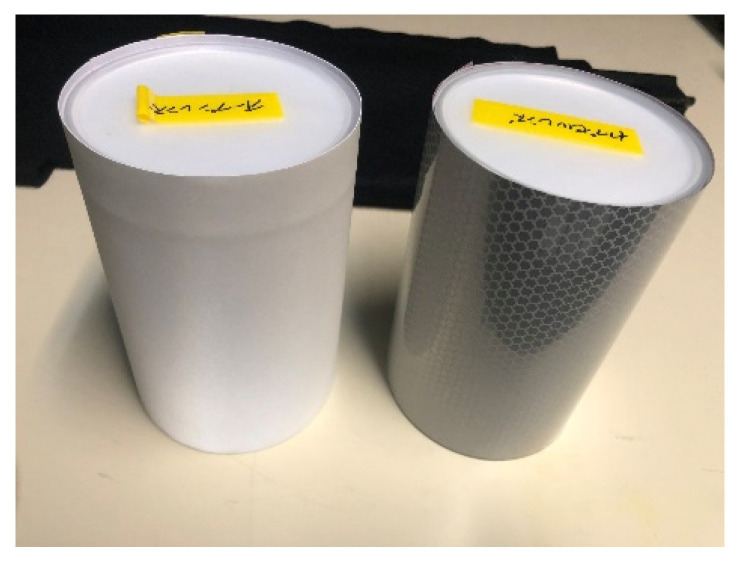
Cylinder with curved surface covered with retroreflective material (left: open lens type, right: encapsulated lens type).

A 10 cm diameter cylinder with sides covered with a retroreflective material was used as the projection target. Figure 5 shows the cylinders used in this study. In the experiment, a combination of open and encapsulated reference points selected in Section 3 was used to illuminate the cylinder from the observation position with a color-calibrated projection light so that the color of the side surface of the cylinder was uniform. Seven colors (red, green, blue, cyan, magenta, yellow, and white) from X-Rite’s ColorChecker under the CIE standard illuminant D65 (ideal source) were used for verification. For both the open and encapsulated models, a total of eight samples were used for comparison: six samples of the proposed method, color calibration assuming Lambert reflection, projected light adjusted to the reflection intensity at the frontal measurement angle of 0°, and no other calibrations. Images of the color-calibrated projection light projected onto each material were captured by a camera, and the pixel changes on the side of the cylinder in the captured images were compared using standard deviation and frequency analysis. In addition, a comparison of the color calibration accuracy was made at two locations on the side of the cylinder based on the color difference from the seven colors of the ColorChecker.

### 4.2. Color Reproduction Accuracy

As discussed in Section 1, color calibration for a given fixed angle can be achieved by creating an LUT between the projector RGB and the reflected color XYZ using conventional methods. We do not claim any novelty in this calibration method. Before validating our proposed method of inferring XYZ for different angles from a small number of angle measurements, we checked how accurate the calibration is with a conventional LUT. In this subsection, we confirm the accuracy when irradiated from the front with an incident angle of 0 degrees.

Table 2 shows the CIE 1976 Delta E color difference between the target colors in ColorChecker and the reproduced colors on the retroreflective materials. There is no significant difference in reproduction accuracy in either material and it can be confirmed that color reproduction is possible with a color difference of around 2. It should be noted that retroreflective materials are highly directional, and slight deviations in observation angle can significantly affect color reproduction.

### 4.3. Verification for Changes in Incidence Angle

**Figure 6:**
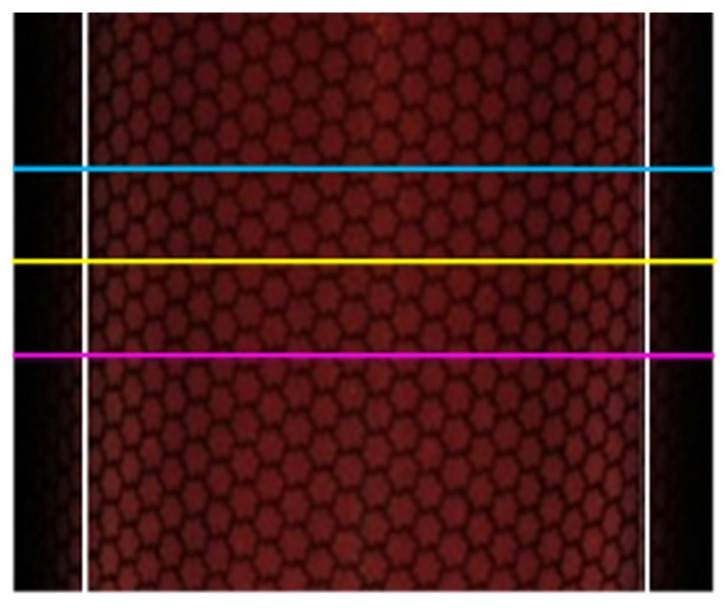
Example of captured image and verification position (horizontal lines between white vertical lines).

**Figure 7:**
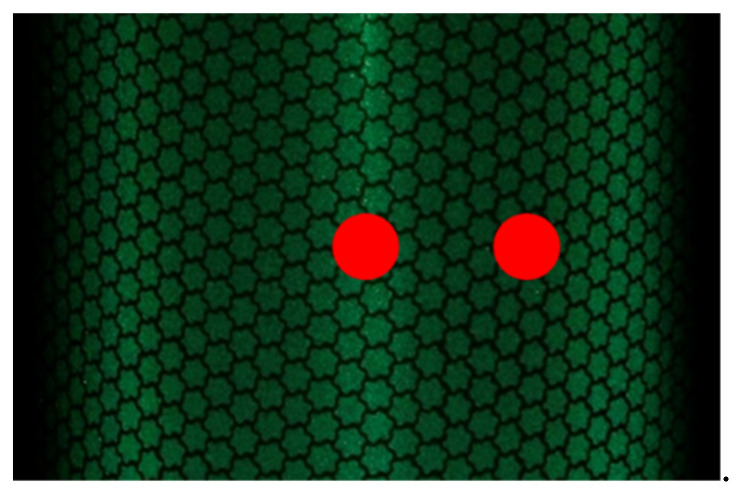
Example of color difference measurement position (red dots).

Figure 6 shows an example of the validation position of the captured image. Standard deviation and frequency analyses were performed by averaging the pixel values of the three horizontal lines in the range of the vertical white lines in Figure 6, and then averaging the average pixel change. Figure 7 shows an example of the color difference measurement position. One of the goals in calibrating retroreflective materials with different reflection intensities at each incidence angle is to obtain uniform RGB values regardless of the incidence angle. To verify the accuracy, the standard deviation is defined as the degree to which the RGB values measured by the camera vary spatially. The one-dimensional variation in RGB values on a specific line on the material is also defined as frequency analyses. The standard deviation should be small, and the frequency analyses should have high power at low frequencies. As shown by the red dots in Figure 7, the color difference was measured at two locations: at the center of the cylinder, where care was taken not to include highlights, and at the midpoint between the center and edge of the cylinder.

**Figure 8:**
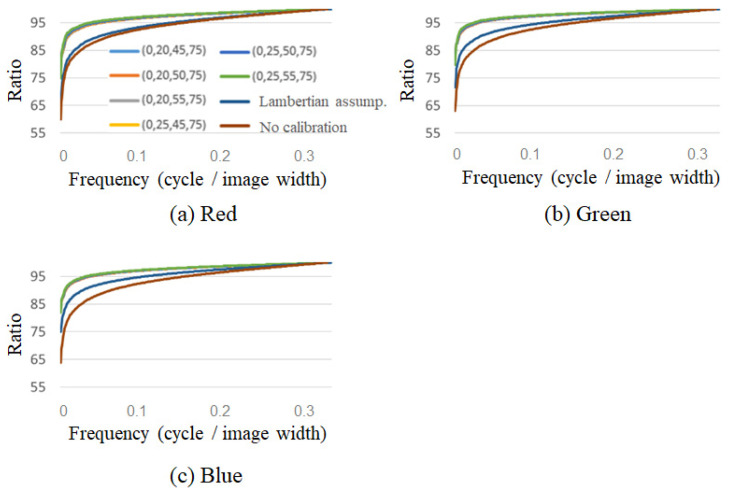
Frequency analysis of captured RGB values on the horizontal line indicated in Figure 6 (green projection onto encapsulated lens type).

**Figure 9:**
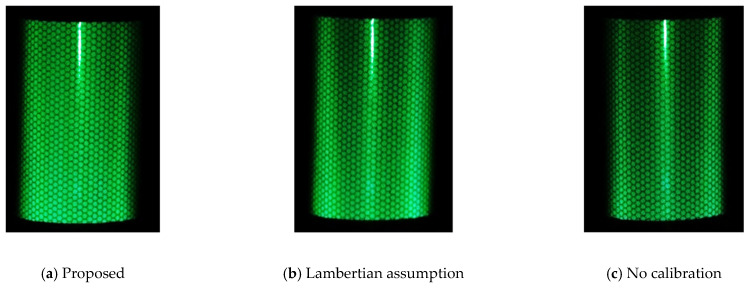
Comparison of visibility by green projection (encapsulated lens type).

Each projected light on a cylindrical surface was photographed using a digital camera, and the standard deviations of the reflected light and frequency analyses were com-pared. Specifically, we compared and verified the standard deviation of the pixel change in the three horizontal lines within the vertical white line shown in Figure 6, and the results of the frequency analysis of the average of the three lines. As an example, Table 3 shows the standard deviation for the projection of green light on the encapsulated lens type, and Figure 8 shows the frequency analysis for each RGB component. The vertical axis shows the percentage of the normalized cumulative power. Figure 9 also shows the projection results of the proposed method on the capsule cylinder, the projection results when the Lambert reflection was assumed, and the projection results without calibration.

Figure 9 shows that the projection result using the proposed method has a higher percentage of low-frequency components and a flatter composition. Table 3 shows that among the proposed methods, the combination of (0°, 20°, 40°, 50°) in particular shows the least variation in pixel values. This indicates that the encapsulated lens type can achieve proper color calibration by interpolating the data measured by the (0°, 20°, 40°, 50°) reference points.

**Figure 10:**
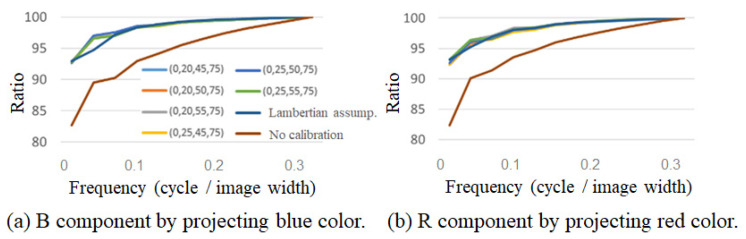
Comparison of cold and warm colors (green projection onto open lens type).

The open lens type was less effective for calibration when using the proposed method. Table 4 lists the standard deviation of the acquired pixel values when projecting green onto an open-type cylinder. It can be seen that there is less difference in the calibration method compared to the encapsulated lens type. This can be attributed to the fact that the reflection characteristics of the encapsulated lens type vary greatly with the angle, whereas those of the open lens type vary little with the angle, as shown in Figure 3a,b. Because the accuracy of the calibration was different for cold and warm projected light, the cumulative power components of the main B component of blue and the main R component of red are shown in Figure 10 as examples. As shown in Figure 10a, for cold colors, the color calibration using the proposed method shows a higher percentage at lower frequencies than the calibration assuming Lambert reflection or no calibration, indicating that the cylindrical surfaces are projected uniformly in space. However, as shown in Figure 10b, for warm colors, the color calibration results using the proposed method do not differ from the results assuming Lambert reflection. This is because the value of the R component is larger than that of the G and B components during the creation of the projected light, resulting in a larger error in the interpolation part. This is thought to have made it difficult to obtain a difference in the warm color system, where R is the main component.

**Figure 11:**
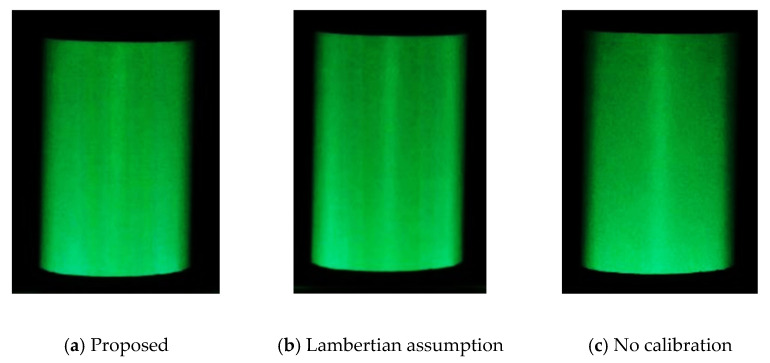
Comparison of visibility by green projection (open lens type).

A comparison of the standard deviation and percentage of low-frequency components in the frequency analysis indicates that measurements with reference points at (0°, 25°, 50°, 75°) or (0°, 25°, 55°, 75°) are relatively better at representing the reflection characteristics. Figure 11 shows the results of the projection of the proposed method (0°, 25°, 50°, 75°), the method assuming Lambertian reflection, and the uncalibrated method on an open cylinder. Figure 11 shows that (b) produces thin light and dark stripes, and (c) has a bright center and a darker surrounding area, whereas the result of the proposed method (a) is close to uniform. This indicates that the proposed color calibration method is useful, although its effect varies depending on the color.

## 5. Conclusions

In this study, we aimed to simplify the color calibration of a projector using retroreflective projection technology (RPT) on a retroreflective material with a known 3D shape and structure under the condition that the projector is observed from the same direction as the light source. First, the reflective properties of the three types of retroreflective materials were measured, and it was confirmed that the open and encapsulated lens types had reflective properties different from the Lambertian reflection. Next, the optimal number of measurements and measurement angles were determined by AIC with the aim of obtaining the reflective properties of the open and encapsulated lens types in a small number of measurements. Based on the data, calibration was performed to obtain a uniform view of the cylinder sides, and the accuracy of the representation of the reflectance characteristics was compared based on the standard deviation of the pixel change in the calibration range and the frequency analysis of the captured images. The results showed that the encapsulated lens type was highly effective. The open lens type was also confirmed to be effective, although the effect was smaller than that of the encapsulated lens type. These results are in general agreement with those of AIC, and it was found that the angle of measurement could be narrowed down by AIC.

The proposed method simplifies the color calibration process for each projection de-vice when the reflective material of interest is known. Because the reflective properties of reflective materials with the same structure are considered to be similar, color calibration of unknown projectors can be performed for the three types of reflective materials by measuring the angles derived in this study. Therefore, for new types of materials with unknown reflective properties, it is necessary to precisely measure the reflective properties once and then use AIC to derive the optimal number of measurements and measurement angle data. However, once the measurement angles have been derived, the color calibration of the same type of reflective material can be easily performed.

In this study, we simplified the color calibration of retroreflective materials when the reflective properties of the projected object are known and when the side of the projected light source changes. Additionally, we set the condition that the object should be observed in the same direction as the light source. It is necessary to verify a method that does not restrict the light source and observation position in the future because it is thought that more effective projection can be achieved by knowing the reflection characteristics when the object is observed from a direction other than that of the light source. Furthermore, the divergence angle of projection light may affect the calibration results. We have to consider this issue in more exacting color reproduction.

## Figures and Tables

**Table 1:** *AIC* at each number of reference angle *k* in Equation (1) for open and encapsulated lens types. A smaller AIC value statistically means that a smaller approximation error is obtained using a smaller number of reference angles.

	Three Angles	Four Angles	Five Angles	Six Angles
Open lens type	−90.39	−118.78	−104.44	−90.41
Encapsulated lens type	−49.69	−57.52	−53.27	−43.27

**Table 2:** Color reproduction accuracy using CIE 1976 Delta E color difference for open and encapsulated lens types.

	Red	Green	Blue	Cyan	Magenta	Yellow	White
Open lens type	2.87	2.89	1.53	0.71	1.98	2.21	1.82
Encapsulated lens type	2.06	1.77	2.13	2.17	2.59	2.00	2.24

**Table 3:** Standard deviation of captured RGB values on the calibrated area (green projection onto encapsulated lens type).

	R	G	B
(0, 20, 40, 55)	29.69	28.72	27.40
(0, 20, 45, 55)	30.40	29.48	28.33
(0, 20, 50, 55)	32.97	31.80	28.52
(0, 25, 40, 55)	29.67	29.21	27.71
(0, 25, 45, 55)	31.44	30.33	28.41
(0, 25, 50, 55)	36.73	35.07	30.29
Lambertian assumption	34.09	34.25	30.49
No calibration	38.55	39.53	40.43

**Table 4:** Standard deviation of captured RGB values on the calibrated area (green projection onto open lens type).

	R	G	B
(0, 20, 40, 55)	3.76	5.01	3.98
(0, 20, 45, 55)	3.24	4.79	3.65
(0, 20, 50, 55)	3.49	4.70	3.64
(0, 25, 40, 55)	3.46	4.90	3.73
(0, 25, 45, 55)	3.25	4.72	3.60
(0, 25, 50, 55)	3.52	4.94	3.85
Lambertian assumption	3.55	5.13	4.06
No calibration	4.28	7.15	5.12

## Data Availability

Data are contained within the article.
